# Raf kinase inhibitory protein reduces bradykinin receptor desensitization

**DOI:** 10.1111/jnc.15614

**Published:** 2022-05-08

**Authors:** Samuel B. Chivers, Allison Doyle Brackley, Nathaniel A. Jeske

**Affiliations:** ^1^ Departments of Oral & Maxillofacial Surgery University of Texas Health San Antonio San Antonio Texas USA; ^2^ Physiology University of Texas Health San Antonio San Antonio Texas USA; ^3^ Pharmacology University of Texas Health San Antonio San Antonio Texas USA

**Keywords:** bradykinin, calcium, GRK2, PKC, RKIP

## Abstract

Inflammatory hyperalgesia represents a nociceptive phenotype that can become persistent in nature through dynamic protein modifications. However, a large gap in knowledge exists concerning how the integration of intracellular signaling molecules coordinates a persistent inflammatory phenotype. Herein, we demonstrate that Raf Kinase Anchoring Protein (RKIP) interrupts a vital canonical desensitization pathway to maintain bradykinin (BK) receptor activation in primary afferent neurons. Biochemical analyses of primary neuronal cultures indicate bradykinin‐stimulated PKC phosphorylation of RKIP at Ser153. Furthermore, BK exposure increases G‐protein Receptor Kinase 2 (GRK2) binding to RKIP, inhibiting pharmacological desensitization of the BK receptor. Additional studies found that molecular RKIP down‐regulation increases BK receptor desensitization in real‐time imaging of primary afferent neurons, identifying a key pathway integrator in the desensitization process that controls multiple GRK2‐sensitive G‐protein coupled receptors. Therefore, RKIP serves as an integral scaffolding protein that inhibits BK receptor desensitization.

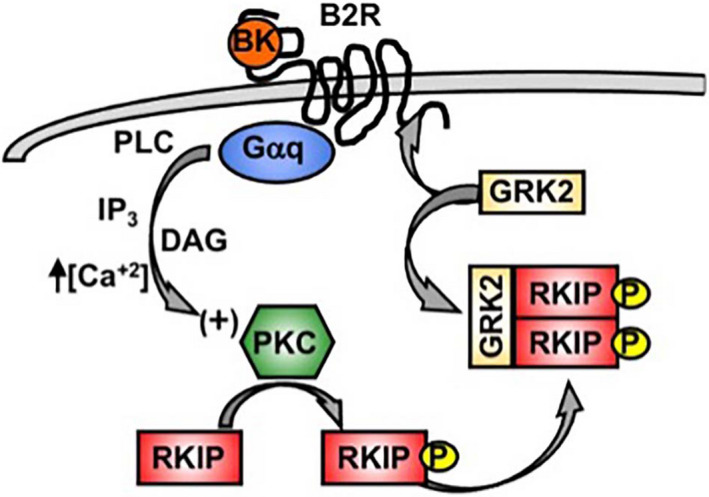

## Graphical abstract

In this study, we investigated unknown biochemical modulators that may contribute to persistent inflammation. We identified that small, physiological concentrations of bradykinin can stimulate protein kinase C (PKC) activity, to increase phosphorylation of Raf Kinase Inhibitory Protein (RKIP) and its association with GPCR Receptor Kinase 2 (GRK2). Experimental results indicate that the scaffolding protein RKIP coordinates GRK2 availability for GPCR desensitization in sensory neurons. Our findings identify a new role for RKIP in regulating inflammatory GPCR responsivity.

**Abbreviations**
Ab: antibody
ANOVA: analysis of variance
B1R: bradykinin type‐1 receptor
B2R: bradykinin type‐2 receptor
BK: bradykinin
BLAST: basic local alignment search tool
CAP: capsiacin
CO2: carbon dioxide
Co‐IP: co‐immunoprecipitation
DAG: diacylgylcerol
DAPI: 4′,6‐diamidino‐2‐phenylindole
DOR: delta opioid receptor
DRG: dorsal root ganglia
DTT: dithiothreitol
ECL: enhanced chemiluminescence
EDTA: ethylenediaminetetraacetic acid
EGTA: egtazic acid
EP4: E‐type prostanoid receptor
FITC: fluorescein isothiocyanate
GFX: GF‐109203X
GPCR: G‐protein coupled receptor
GRK2: GPCR receptor kinase 2
Gα12/13: G‐protein alpha subunit 12/13
Gαi: G‐protein alpha subunit i
Gαq/11: G‐protein alpha subunit q/11
Gαs: G‐protein alpha subunit s
HEPES: (4‐[2‐hydroxyethyl]‐1‐piperazineethanesulfonic acid)
IACUC: Institutional Animal Care and Use Committee
ICC: immunocytochemistry
IHC: immunohistochemistry
IP3: inositol triphosphate
KCl: potassium chloride
MOR: Mu opioid receptor
mRNA: messenger ribonucleic acid
NGF: nerve growth factor
NIH: National Institutes of Health
PB: phosphate buffer
PBS: phosphate buffered saline
PDBu: phorbol 12,13‐dibutyrate
PGE2: prostaglandin E2
PKA: protein kinase A
PKC: protein kinase C
PVDF: polyvinyl difluoride
RKIP: Raf kinase inhibitory protein
SDS‐PAGE: sodium dodecyl sulfate polyacrylamide gel electrophoresis
SEM: standard error of the mean
Ser153: serine 153
SES: standard extracellular solution
siRNA: small interfering ribonucleic acid
SOCE: store operated calcium entry
TBS‐T: tris buffered saline – 0.1% tween
TG: trigeminal ganglia
WB: western blot

## 1 INTRODUCTION

Inflammatory hyperalgesia following tissue injury occurs as afferent nerve fibers become sensitized to physical and chemical stimuli. Inflammatory mediators such as bradykinin (BK) activate receptors on afferent nerve endings (Dray, 1995) to reduce the activation threshold. Despite rapid canonical desensitization mechanisms for G‐protein coupled receptors (GPCRs) such as the bradykinin type‐2 receptor (B2R) (Leeb‐Lundberg et al., 2005), patients often experience persistent inflammatory sensitization following acute injury. This phenomenon has been understudied and is an important therapeutic target for patients experiencing persistent pain syndromes. The research herein identifies a scaffolding molecule that prolongs bradykinin receptor desensitization in primary afferent neurons and may serve some clinical utility.

G‐protein Receptor Kinase (GRK) is a multi‐domain enzyme expressed throughout mammalian tissues and is critically involved in G‐protein association and GPCR desensitization. Several isoforms, including GRKs 2, 3, 5, and 6, phosphorylate GPCRS to stimulate β‐arrestin association and receptor internalization (Kelly et al., 2008). GRK2 has been previously characterized as the primary isoform responsible for dictating bradykinin and beta‐adrenergic receptor desensitization in multiple cardiac pathology models (Gambardella et al., 2020; Izzo et al., 2008; Santulli et al., 2011; Sorriento et al., 2015). Yet, multiple non‐GPCR proteins also contribute to the GRK2 interactome (Penela et al., 2010), including calmodulin and p38 (Patial et al., 2009; Penela et al., 2003), thereby complicating GRK2 functions in GPCR receptor signal management. Indeed, GRK2 association with the scaffolding protein Raf Kinase Inhibitory Protein (RKIP) inhibits desensitization of the β‐adrenergic receptor (Maimari et al., 2019).

RKIP serves as a multi‐function scaffolding protein capable of serving as a “sink” for free cytosolic GRK2. RKIP phosphorylation at Ser153 by Protein Kinase C stimulates RKIP dimerization that can then bind to GRK2 and remove it from targeting other substrates, including GPCRs (Deiss et al., 2012; Lorenz et al., 2003). We hypothesized that a similar scenario could inhibit B2R desensitization in a more physiologically‐relevant model. Experimental results herein indicate that RKIP binding to GRK‐2 actively dictates B2R desensitization in primary afferent neuronal cultures, potentially contributing to the persistent nature of clinical inflammatory pain.

## 2 METHODS

### 2.1 Animals and materials

Procedures utilizing animals were approved by the University of Texas Health San Antonio's (UTHSA) Institutional Animal Care and Use Committee (IACUC, protocol 20130050AR, approved 04/20). Studies were conducted in accordance with the policies for the ethical treatment of animals established by the National Institutes of Health (NIH) with every effort made to limit animal discomfort and the number of animals used. Sprague Dawley rats (3–4 weeks of age, 50–75 g; Charles River Laboratories, RRID:RGD_10395233) were used throughout this study. Animals were housed in clean, Allentown Static cages, 3/cage, with a 12 h light/dark cycle with food and water ad libitum before use. Unless otherwise stated, common chemical reagents were purchased through Sigma–Aldrich.

### 2.2 Primary cultures

For biochemistry, trigeminal ganglia (TG) were dissected bilaterally from adult male Sprague–Dawley rats following decapitation (3 rats [6 TGs] per plate). TG were dissociated by collagenase treatment (30 min; Worthington, Cat # LS004193), followed by trypsin treatment (30 min; Gibco, Cat# 25300–054). Dissociated TG were re‐suspended in complete media (Dulbecco's modified Eagle's medium, Gibco, Cat# 11960–044) supplemented with 10% fetal bovine serum (FBS; Gibco, Cat#10437–028), 100 ng/ml nerve growth factor (NGF; Harlan Laboratories, Cat# 5023), mitotic inhibitors (SigmaAldrich, Cat# F6627, 1% penicillin/streptomycin (Gibco, Cat# 15070–063), and 1% glutamine (Gibco, Cat# 25030–081) and plated on poly‐d‐lysine‐coated plates (Corning, Cat# 354595). Similarly, for functional Ca^2+^ imaging studies, TG was dissociated by 40 min co‐treatment with collagenase and dispase II (Sigma–Aldrich, Cat# D4693). Cells were then re‐suspended in complete media and plated on poly‐d‐lysine/laminin‐coated coverslips (BD Biosciences, 6 coverslips/rat [2 TGs or 6 DRGs], Cat# 354087 Cultures were maintained at 37°C and 5% CO_2_ and grown for 1–2 days for functional studies or 5–6 days for biochemistry. Media was changed the day following initial culturing and every 2 days thereafter. TG were utilized for biochemical experiments to satisfy NIH requirements to reduce animal use in research.

### 2.3 Immunohistochemistry and immunocytochemistry

Male Sprague–Dawley rats were anesthetized with an intraperitoneal (*i.p*.) injection of pentobarbital (100 mg/kg) and were transcardially perfused with 100 ml 0.9% saline in water followed by 250 ml of fixative (4% paraformaldehyde in 0.1 M phosphate buffer [PB] at pH 7.4). Dorsal root ganglia (DRG) were dissected bilaterally at L4‐L6 and left intact. Tissue specimens were rinsed in 0.1 M PB for 10 min three times before rinsing in 0.1 M PB with 15% sucrose for 1 h at 22°C. Intact DRG were rocked at 4°C in 30% sucrose in 0.1 M PB for 18 h. Cryoprotected ganglia were embedded in Neg‐50 mounting medium (Richard‐Allan Scientific, Kalamazoo, MI) and a cryostat was used to slice structures into 30‐μm transverse sections placed onto Superfrost Plus slides (Fisher Scientific, Cat# 22‐037‐246). Processed slides were dried and stored at −20°C. Cultured rat TG neurons were grown on poly‐D lysine coated coverslips for 2–3 days in normal media. Coverslips were rinsed with phosphate‐buffered saline (PBS) and fixed with 4% paraformaldehyde in 0.1 M PBS for 10 min at 25°C. Following fixation, coverslips were rinsed twice with PBS and incubated with 5% normal goat serum (Gibco, Cat# PCN5000, 0.5% Triton X‐100 in PBS for 30 min at 25°C.

Staining steps were performed at 22°C. Slides were rinsed in 0.1 M PBS three times for 10 min each. Samples were then incubated for 90 min in blocking solution (2% bovine ‐globulin (Sigma–Aldrich, Cat# 345876), 4% normal horse serum (Sigma–Aldrich, Cat# H0146), and 0.3% Triton X‐100 (Fisher Scientific) in 0.1 M PBS) to minimize non‐specific binding. Next, slides and coverslips were incubated in primary antibody against RKIP (Cat# 07–137, Millipore; at 1:100 dilution in blocking solution) for 18 h in a humidifier. Antibody specificity was validated with no immunoreactivity in RKIP knockout mice (Subramanian et al., 2014). Slides and coverslips were also incubated with a primary antibody specific for B2R (Clone 20/B2, BD Transduction Laboratories, at 1:100 dilution in blocking solution) and TOPRO and DAPI, as indicated and performed previously (Duchene et al., 2002).

Next, tissue was rinsed with 0.1 M PBS and placed in a dark humidifier for 90 min to incubate in anti‐rabbit secondary‐linked Alexa Fluor 488 (Molecular Probes at 1:100 dilution in blocking solution, to visualize RKIP immunoreactivity) and anti‐mouse secondary‐linked Alexa Fluor 594 (Molecular Probes at 1:100 dilution in blocking solution, to visualize B2R immunoreactivity). Nuclei marker TOPRO (Invitrogen; 1:5000 dilution) or DAPI (ThermoFisher; 1:5000 dilution) were added to the secondary antibody‐blocking solution. The tissue was rinsed in 0.1 M PBS, followed by a final wash in deionized water. Once dry, stained DRG slides were coverslipped with Vectashield (Vector Labs), and stored at 4°C until they were evaluated by confocal microscopy. Coverslips were attached to Superfrost Plus slides, sealed, and stored at −20°C until confocal evaluation. Images were obtained using a Nikon 90i microscope equipped with a C1si laser scanning confocal imaging system. Confocal images are representative of four individual trials.

### 2.4 TG harvesting and Co‐Immunoprecipitation (Co‐IP)

On identified days and following specific treatments, cultures were rinsed twice with ice‐cold PBS, brought down in Co‐IP buffer (20 mM HEPES, 120 mM NaCL, 20 mM NaF, 20 mM 2‐glycerol phosphate, 1 mM EDTA, 1 mM EGTA, 1 mM DTT, 1 mM orthovanadate, 0.1% Triton X‐100, 1 mM benzamidine, pH to 7.45), lysed by 20 passes through a 25 g needle, and incubated on ice 15 min prior to clearing by centrifugation (500 μg, 1 min) and subsequent Bradford (Bradford, 1976) analysis of remaining supernatant for protein concentrations. Equal amounts of protein (500 μg) were immunoprecipitated with 1 μg anti‐GRK2 (clone C‐15, Santa Cruz Biotechnology) with GRK2 antibody specificity previously confirmed in inducible GRK2 sensory neuron knockout animals (Wang et al., 2011). 50 μg samples were kept for whole cell lysate analysis and confirmation of targeted protein expression. 500 μg samples were incubated with GRK2 Ab 18 h at 4°C on a shaking rack set to 180 rpm. Protein‐A agarose beads (Abcam, Cat# ab193254) were incubated with 56°C‐treated bovine serum albumin for the same time period under similar conditions. Following primary antibody incubation, samples and beads were allowed to precipitate by gravity on ice, upon which samples and beads were combined and incubated at 4°C shaking for 2 h. Following this, samples were rinsed 4 times with Co‐IP buffer and combined with SDS‐PAGE loading buffer for WB analysis.

### 2.5 Western blot (WB) analysis

Cleared TG lysate and Co‐IP samples were resolved by 15% sodium dodecyl sulfate polyacrylamide gel electrophoresis (SDS‐PAGE) and transferred to polyvinyldifluoride membranes (PVDF; Millipore). Membranes were blocked with 5% non‐fat dried milk in Tris‐buffered saline/Tween 20 (TBS‐T: 15.35 mM Tris/HCl, 136.9 mM NaCl, pH 7.6, with 0.1% Tween 20) or 5% bovine serum albumin (Sigma) in TBS‐T containing phosphatase inhibitor sodium orthovanadate (1 μM) for phosphorylation‐specific antibodies. WBs were visualized using anti‐GRK2 (clone C‐15, Santa Cruz Biotechnology), anti‐phospho‐Ser153 RKIP (Abcam, clone EP2845Y), anti‐total RKIP (Millipore, clone 4A11), or anti‐β‐actin^18^ (Santa Cruz Biotechnology, clone I‐19‐R), followed by appropriate horseradish‐peroxidase‐conjugated secondary antisera (GE Healthcare) and ECL (enhanced chemiluminescence or prime) detection following the manufacturer's protocol. Antibody specificities were verified by the manufacturers, BLAST sequence analysis, and in previous publications as indicated. Integrated density measurement values, equivalent to the product of area and mean gray value by histogram analysis, were performed using NIH ImageJ software.

### 2.6 PKC kinase activity

PKC activity was assessed in TG neurons treated with BK or PDBu (as indicated). Immediately following treatment, TG were collected in ice‐cold PBS, washed twice with PBS, and suspended in 1X sample preparation buffer (50‐ mM Tris–HCl, 10 mM benzamidine, 5 mM EDTA, 10 mM EGTA, pH 7.5). Samples were sonicated five times for 10 s each and centrifuged at 100 000 *g* for 60 min at 4°C. The resulting supernatant was collected and quantified by Bradford analysis. Aliquots (50 μg) of sample supernatants were assayed for PKC activity following the manufacturer's instructions (CycLex PKC Super Family Kinase Assay Kit, MBL International Corporation, Cat#CY‐1175). The PKC substrate consisted of a synthetic peptide corresponding to CPI‐17 that was fixed to the microtiter plate. The final ATP concentration at the time of assay was 62.5 μM. Results are representative of 3 independent trials conducted in triplicate. “Independent trial” corresponds to an independent culturing event, whereas samples were quantified in three equal aliquots (‘triplicate’).

### 2.7 Single‐Cell Ca^2+^ imaging

Following 2 h serum‐starvation, cultured TG were loaded with Fura‐2 AM (1 μM; Invitrogen, Cat# F1221) and pluronic F‐127 (0.04%; Invitrogen, Cat# P3000MP) for 1 h at 37°C in the dark, in standard extracellular solution (SES) containing (in mM): 140 NaCl, 4 KCl, 2 CaCl_2_, 1 MgCl_2_, 10 HEPES, 10 D‐(+)‐glucose, pH 7.40. Neurons were observed on an inverted Nikon Eclipse T_i_‐U microscope fitted with a 20x/0.75 numerical aperture Fluor objective and imaged using MetaFluor System for Ratio Fluorescence (MetaMorph). Fluorescent images were taken as previously described (Brackley et al., 2016; Brackley et al., 2017). The following criteria were used to indicate positive sensory neuronal phenotype within a heterogenous culture: [1] bright round cell bodies with clear nuclei (Goldenberg & De Boni, 1983; Liu et al., 2013), [2] depolarization in response to 50 mM KCl (Khasabova et al., 2004; Pettinger et al., 2013), and [3] sensitivity to capsaicin (CAP, 1 μM; 25% above baseline (Brackley et al., 2016; Brackley et al., 2017); Sigma). Corresponding filters were used to restrict the analysis to fluorescein isothiocyanate (FITC)‐labeled small interfering RNA (FITC‐siRNA) or green fluorescent protein (GFP)‐positive TG.

The treatment timeline proceeded as follows: 1 min baseline, 30‐sec BK administration, 5‐min washout, 30‐sec BK administration, 2‐min washout. F340/380 was recorded in 3‐sec bins. CAP and KCl sensitivity were determined after BK responses were recorded, with CAP(−) and/or KCl(−) cells removed from study inclusion. Results represent various n (typically >60 neurons) from >5 coverslips per BK concentration, across TGs from four rats bilaterally harvested and cultured for coverslip use. Positive transfection with RKIP siRNA was determined by FITC(+) expression in neurons.

### 2.8 RKIP siRNA

Specific siRNA and FITC‐labeled siRNA duplexes custom‐designed to target RKIP were designed and ordered from Qiagen. The sequence for the sense strand of RKIP siRNA was 5’‐ACACAGGUCUGCACCGCUAUU‐3′ and the antisense strand of RKIP siRNA was 5’‐UAGCGGUGCAGACCUGUGUUU‐3′.

For experiments utilizing RKIP siRNA, cells were transfected with siRNA or FITC‐labeled siRNA (450 ng/coverslip; 20 μg/10‐cm plate) for 24 h and the transfection.

mix was replaced with complete media for 16 h prior to experimentation. RKIP siRNA duplexes were transfected into cultured sensory neurons using HiPerFect (Qiagen, Cat# 301704), following the manufacturer's directions. Additional cells were treated with FITC‐labeled Ambion negative control scrambled siRNA (FITC(−), Ambion, Cat# 4404021), used as a negative control to determine the specificity of RKIP siRNAs. TG cultures were transfected with siRNA or FITC‐labeled siRNA targeted against RKIP mRNA, or with scrambled siRNA or no siRNA (mock). Following treatment, cells were homogenized in a homogenization buffer containing 1% Triton X‐100 with 20 strokes using a Potter‐Elvehjem pestle and glass homogenizer tube. Homogenates were placed on ice for 15 min incubation and then centrifuged at 1000 *g* for 1 min to remove nuclei and unlysed cells from the homogenate. The resulting supernatant, whole‐cell lysates, were collected and analyzed by WB.

### 2.9 Statistical analyses

Statistical analyses were performed by a different individual from the experimenter and randomization procedures were not required. Data were not assessed for normality and no tests for outliers were performed. Sample sizes were calculated to provide an 80% power at a two‐tailed *p* < 0.05 criteria for significance. Student's *t* tests and one‐way ANOVA were employed to determine statistical significance between identified groups, with p values of <0.05, 0.01, 0.005 used. Bonferroni analyses were used, as indicated, for post hoc determination of ANOVA results. GraphPad Prism is used to calculate power analyses, SEM, and statistical values.

## 3 RESULTS

**FIGURE 1:**
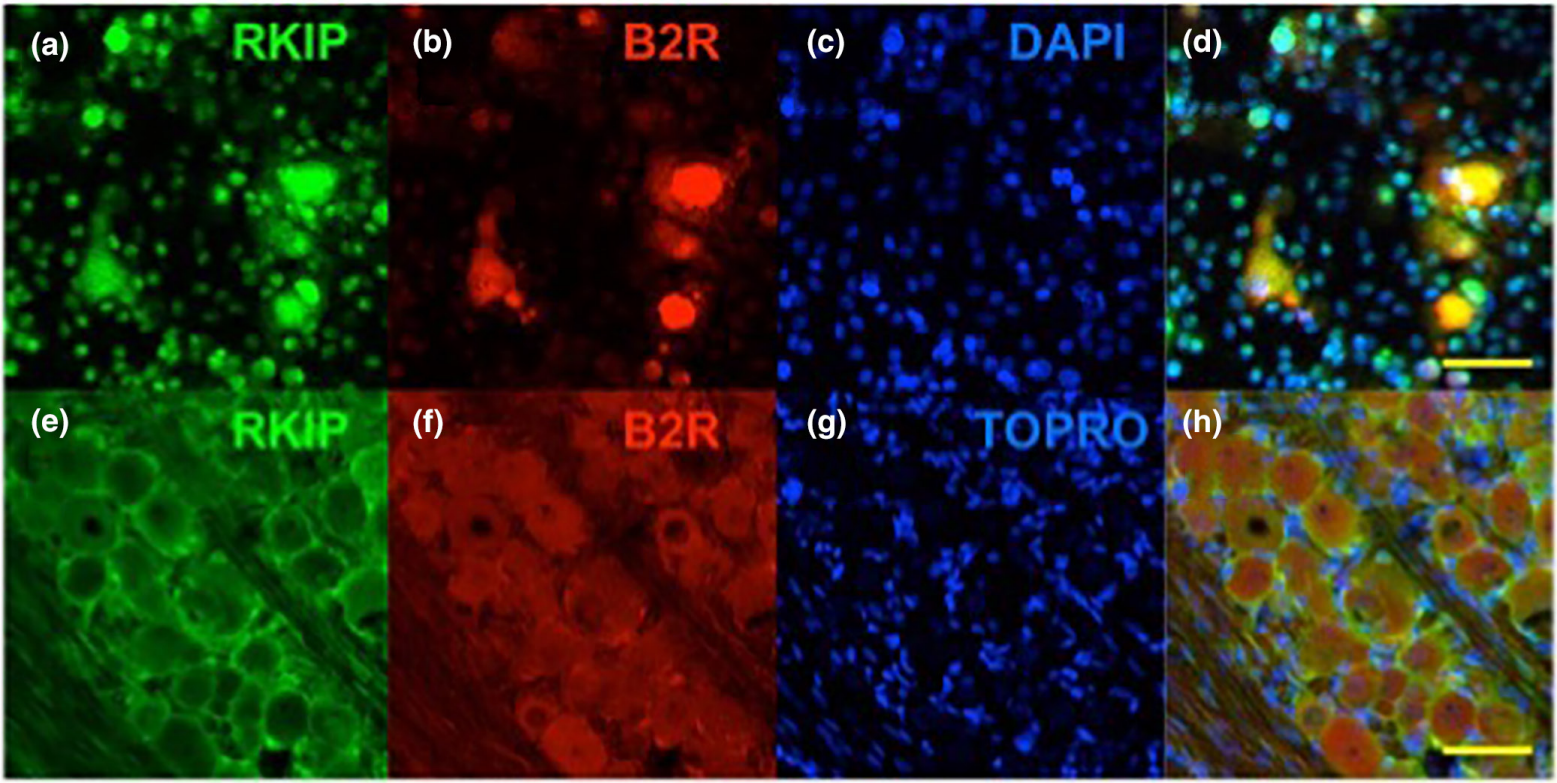
Immunocytochemical (ICC) and immunohistochemical (IHC) co‐localization of RKIP and B2R in peripheral sensory neurons. (a–d) RKIP (green), B2R (red), and nuclear stain DAPI (blue) IHC in cultured trigeminal sensory neurons. (e–h) RKIP (green), B2R (red), and nuclear stain TOPRO (blue) IHC in intact dorsal root ganglia. Results representative of 4 independent animals, yellow bar = 50 mm

Few studies have focused on protein expression patterns of RKIP in peripheral sensory neurons. Therefore, we performed immunocytochemical and immunohistochemical analyses of RKIP and B2R expression in cultured TG and intact DRG neurons. Both neuronal types were studied to determine potential differential expression patterns (Lindquist et al., 2021). In Figure 1, TG neuronal staining identifies strong RKIP (green, Figure 1a) co‐expression with B2R (red, Figure 1b) in a heterogenous primary TG cultures in vitro (Figure 1d). Similarly, RKIP (green, Figure 1e) and B2R (red, Figure 1f) demonstrate strong co‐expression in intact DRG in vivo (Figure 1h). However, anatomical co‐expression does not indicate functional RKIP modulation of BK receptor activity sensory neurons, which is necessary to establish translational relevance to feed‐forward regulation.

**FIGURE 2:**
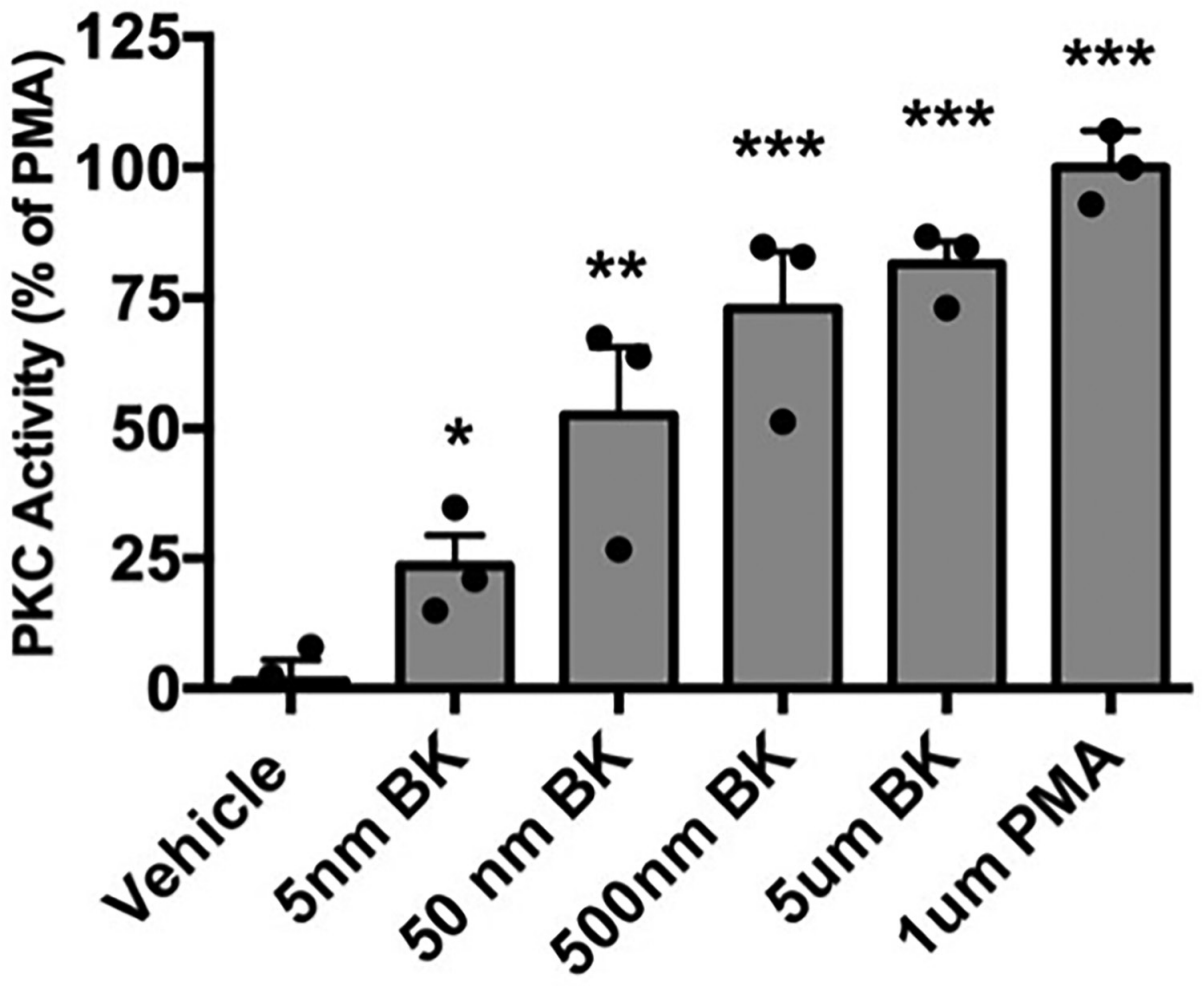
BK activates PKC in sensory neurons. BK (5–5 μM) activate PKC in cultured TG neurons. Mean PKC activities ± SEM shown, *p < 0.05, **p < 0.01, ***p < 0.005 compared to vehicle, *n* = 3 independent primary culture preparations, each in triplicate, one‐way ANOVA with Bonferroni post‐hoc correction (*p* < 0.0001, *F* = 22.31, DF*n* = 5)

Bradykinin is an important peptide that regulates multiple physiological systems. In sensory neurons, it primarily activates B2R to stimulate Gαq signaling mechanisms, including the activation of PKC through IP3‐receptor‐activated intracellular calcium accumulation and diacylglycerol (DAG) generation (Gomez et al., 2011; Jeske et al., 2006; Tippmer et al., 1994; Vellani et al., 2001). Therefore, TG cultures were treated with increasing concentrations of BK ranging from physiological significance (5‐50 nM [Hargreaves & Costello, 1990; Hargreaves et al., 1988b]) to supra‐physiological significance (5 mM) (Hess et al., 1994) to gauge PKC activation. As shown in Figure 2, physiological BK (5 nM) stimulated PKC activity equal to approximately 25% of 1 μM PMA (positive control). PKC activity dose‐dependently increased with BK concentration. Importantly, BK activated PKC in cultured sensory neurons below supraphysiological concentrations used in many in vitro studies (Hess et al., 1994).

**FIGURE 3:**
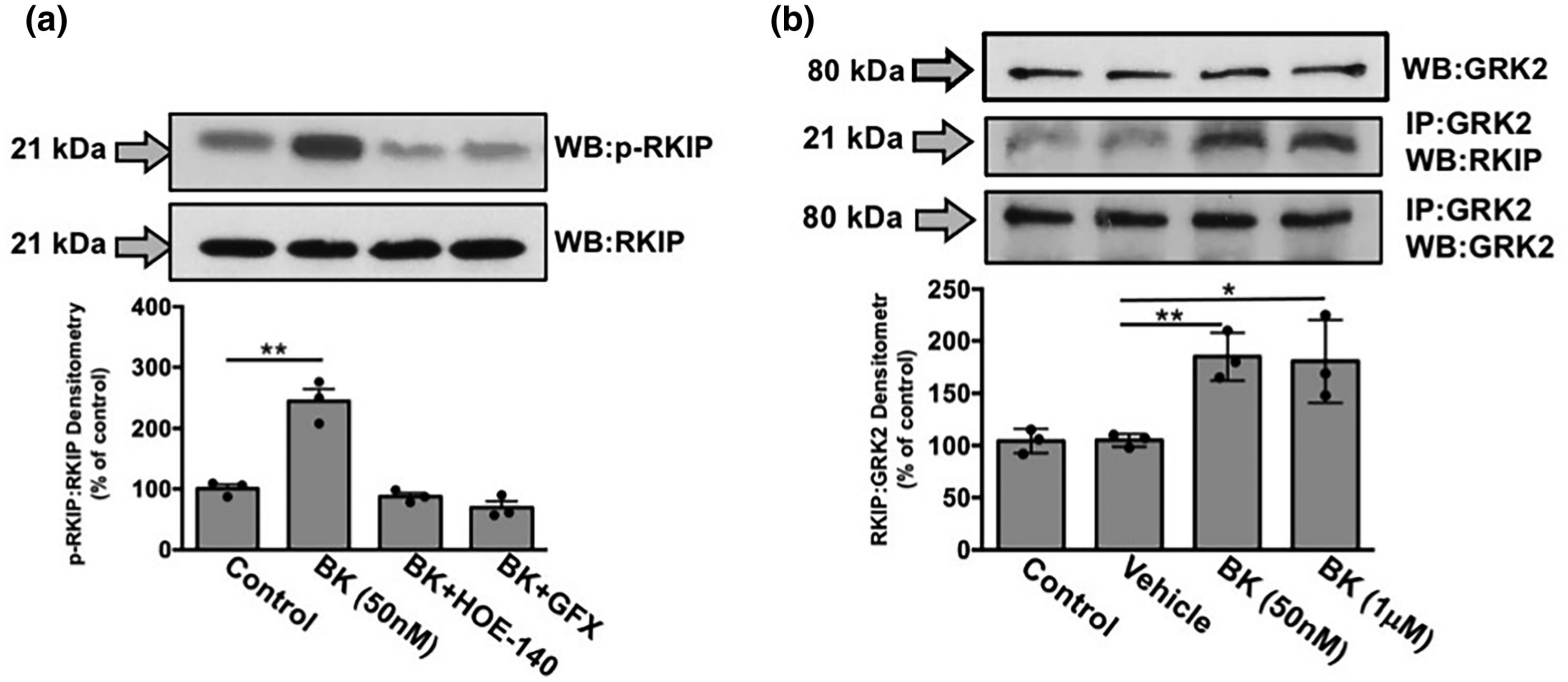
(a) BK phosphorylates RKIP in sensory neurons. Control (ddH_2_O vehicle), BK (50 nM, 5 min), BK plus HOE‐140 (1 μM, B2R antagonist), or BK plus GF 109203X (GFX, 10 μM, PKC inhibitor) treatment of cultured TG neurons. Representative Western blot of cleared lysates probed for phosphorylated RKIP (Ser153) and total RKIP. Mean band densitometries ± SEM shown, **p < 0.01 compared to control, one‐way ANOVA with Bonferroni correction, *n* = 3 independent primary culture preparations/treatment (*p* < 0.0001, *F* = 44.49, DF*n* = 3). (b) BK increases RKIP/GRK2 association in sensory neurons. Control (no treatment), vehicle (ddH_2_O), BK (50 nM or 1 μM, 5 min) treatment of cultured TG neurons. Representative Western blot of GRK2 expression across all treatment conditions (top blot) and GRK2 immunoprecipitates probed for RKIP (co‐IP, middle blot) and GRK2 (to demonstrate equal loading, bottom blot). Mean band densitometries ± SEM shown, *p < 0.05, **p < 0.01 compared to vehicle, one‐way ANOVA with Bonferroni correction, *n* = 3 independent primary culture preparations/treatment (*p* = 0.0035, *F* = 11.51, DF*n* = 3)

RKIP phosphorylation at Ser153 by PKC controls dimerization and subsequent targeting of GRK2 association (Deiss et al., 2012; Lorenz et al., 2003). Therefore, we probed whether BK could stimulate phosphorylation of RKIP at Ser153 in cultured sensory neurons. As demonstrated in Figure 3a, TG cultures were treated with vehicle, 50 nM BK, BK + HOE‐140 (1 μM, B2R antagonist) or BK + GFX (GF109203X, 10 μM, broad‐spectrum PKC inhibitor). Physiological BK (50 nM) increased RKIP phosphorylation twofold in a manner sensitive to B2R antagonism and PKC inhibition. We also performed co‐IPs from cultured sensory neurons treated with physiological BK to investigate RKIP association with GRK2. In Figure 3b, BK (50 nM and 1 μM) increased RKIP Co‐IP with GRK2 compared to vehicle‐treated cultures. Interestingly, more concentrated BK treatment did not increase GRK2 association with RKIP as it did with increasing PKC activity (Figure 2). Also, acute treatment with BK failed to change the endogenous expression of GRK2. This suggests that at a physiological BK, far lower than what is used in many studies, RKIP maximally sequesters GRK‐2 in primary sensory neurons.

**FIGURE 4:**
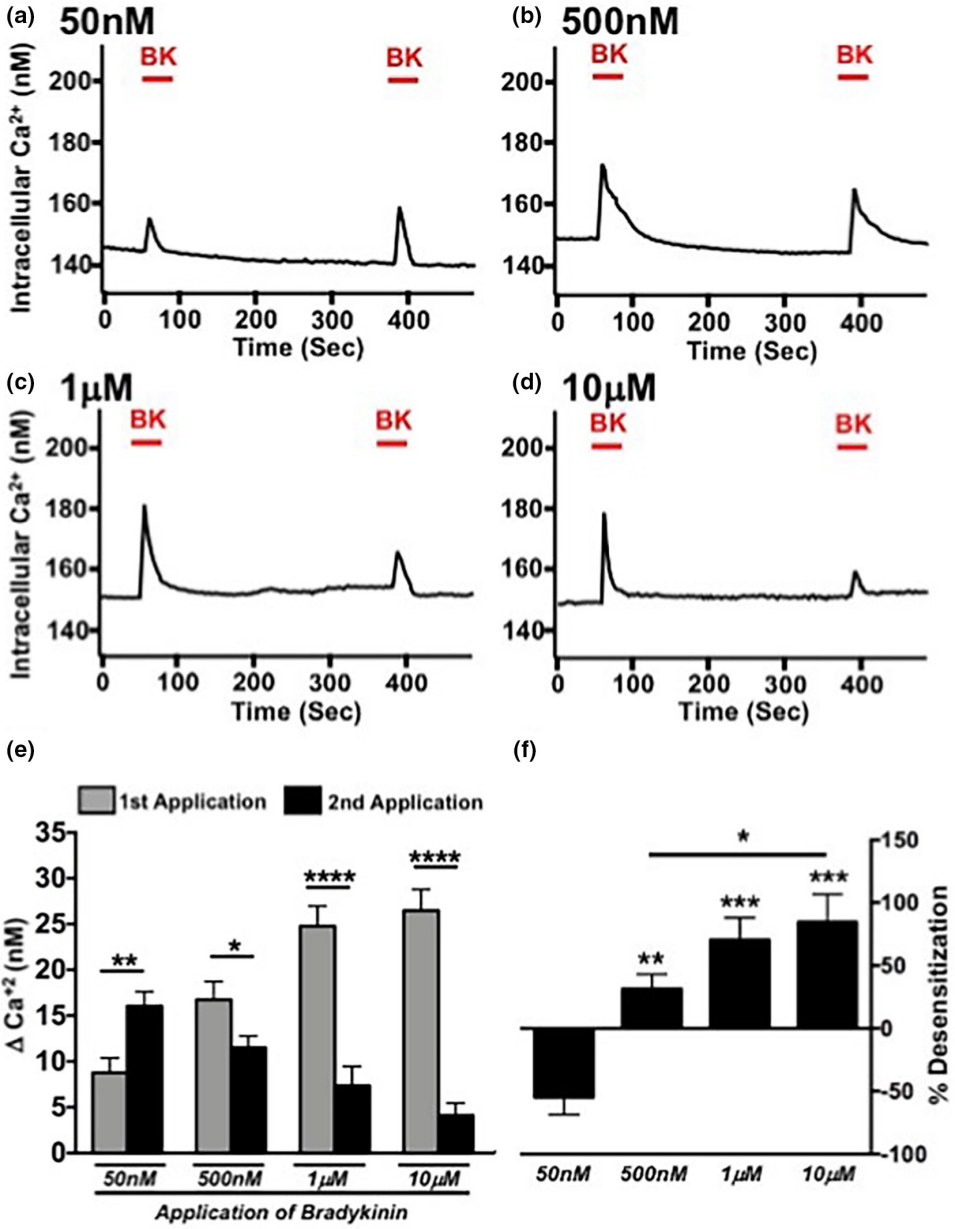
Tachyphylactic BK‐induced calcium response desensitization in sensory neurons. Cover slipped TG neurons exposed to repeated BK at 50 nM (a), 500 nM (b), 1 μM (c), or 10 μM (d) in SES along the following treatment paradigm: 1 min baseline, 30 s BK, 5 min wash, 30 s BK, 1 min wash. Representative real‐time Ca^+2^ imaging curves shown. (e) Cumulative results from individual neurons illustrated for BK at 50 nM (*n* = 67 neurons, **p = 0.0018, *t* = 3.207, df = 134)), 500 nM (*n* = 50 neurons, *p = 0.0293, *t* = 2.213, df = 100), 1 μM (*n* = 56 neurons, ****p < 0.0001, *t* = 5.690, df = 112), 10 μM (*n* = 66 neurons, ****p < 0.0001, *t* = 8.252, df = 112), peak average delta in Ca^+2^ shown ± SEM, T‐test between first and second BK applications. (f) Percent desensitization from first to second BK application calculated for each set of responses, displaying mean values ± SEM, *p < 0.05, **p < 0.01, ***p < 0.005 compared to 50 nM and as shown, one‐way ANOVA with Bonferroni correction (*p* = 0.002, *F* = 14.71, DF*n* = 3)

Canonical GPCR desensitization involves GRK2‐dependent receptor internalization to reduce signal saturation in biological systems (Aragay et al., 1998; Barak et al., 1999; Iacovelli et al., 1996; Neill et al., 1998; Schlador & Nathanson, 1997; Zhang et al., 1998; Zhang et al., 1999). Given that B2R activation of Gαq drives Ca^+2^ accumulation from internal stores (Cruzblanca et al., 1998), we assessed receptor desensitization through real‐time Ca^+2^ imaging following repeated BK administrations at multiple concentrations, as has been demonstrated previously (Bascands et al., 1993). In Figure 4, we identified concentration‐dependent effects on repeated BK‐induced Ca^+2^ responses (Figure 4a, e, f). Supporting a feed‐forward mechanism, a sensitizing effect of BK was observed at the only physiological concentration tested (50 nM). On the contrary, BK receptor desensitization was observed at supraphysiological concentrations (500 nM, 1 μM, and 10 μM). Importantly, Ca^+2^ accumulation curves for individual neurons across all 4 concentrations demonstrate equal rates of accumulation following agonist application. This suggests that nanomolar increases in BK, as occur in inflamed tissue (Hargreaves et al., 1988b; Hargreaves & Costello, 1990), sensitize functional responses in sensory neurons.

**FIGURE 5:**
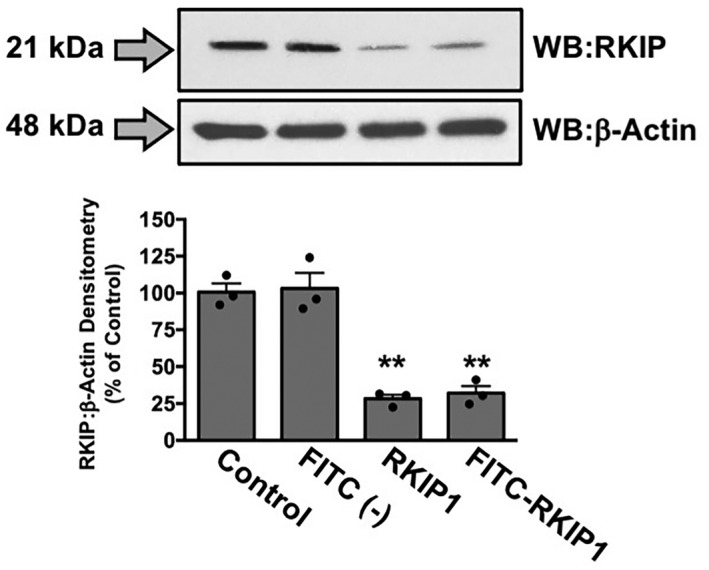
Sirna knockdown of RKIP protein expression. Cultured TG sensory neurons transfected in mock fashion (Hiperfectamine only), with FITC(−) (FITC‐labeled Ambion negative control), RKIP1 siRNA (designed against RKIP), or FITC‐RKIP1 (same sequence as RKIP1, labeled with FITC for identification). Representative Western blot of cleared lysates probed for RKIP and β‐Actin (loading control). Mean band densitometries ± SEM shown, **p < 0.01 compared to control, one‐way ANOVA with Bonferroni correction, *n* = 3 independent primary culture preparations/treatment (*p* = 0.0007, *F* = 0.5070, DF*n* = 3)

**FIGURE 6:**
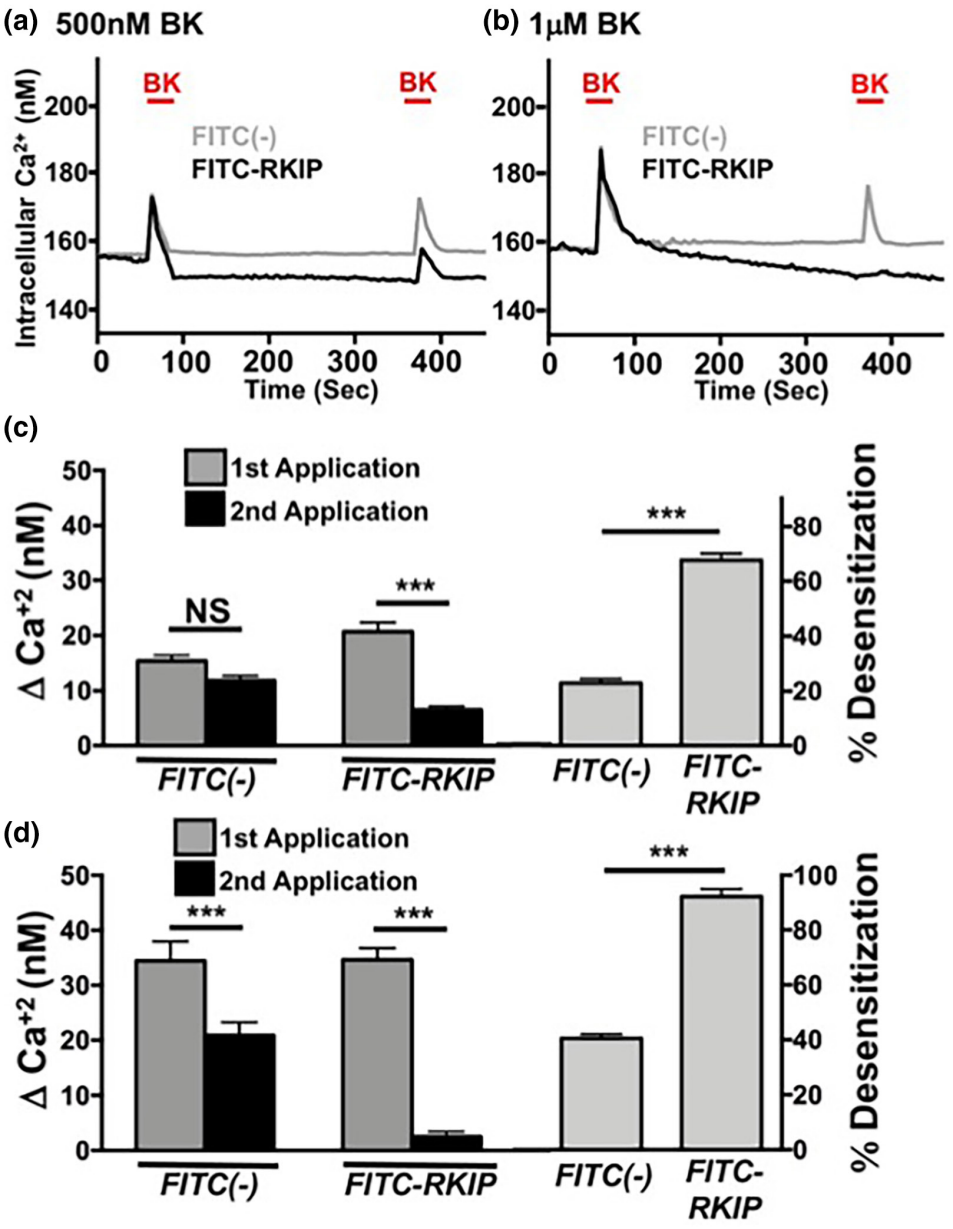
RKIP siRNA increases BK tachyphylactic desensitization in sensory neurons. Cover slipped TG neurons exposed to repeated BK 500 nM (a) or 1 μM (b) in SES following the treatment paradigm in Figure 4, following FITC‐labeled Ambion negative control (FITC(−) transfection (gray line) or transfection with FITC‐RKIP1 siRNA (black line). Representative real‐time Ca^+2^ imaging curves shown. Cumulative results (peak average delta in Ca^+2^ shown ± SEM) and percent desensitization from neurons treated with BK at 500 nM (c, FITC(−) *n* = 49, NS = no significance, *t* = 1.748, df = 98, FITC‐RKIP *n* = 61 neurons, ****p < 0.0001, *t* = 7.586, df = 122, mock vs FITC‐RKIP %desensitization ****p < 0.0001, *t* = 11.31, df = 110) or 1 μM (d, FITC(−) *n* = 41 neurons, ****p < 0.0001, *t* = 5.480, df = 82, FITC‐RKIP *n* = 63 neurons, ****p < 0.0001, *t* = 13.80, df = 126, mock vs FITC‐RKIP %desensitization ****p < 0.0001, *t* = 4.419, df = 104), T‐test between groups as shown

We next sought to investigate whether RKIP expression functionally enhances BK responses in sensory neurons. Transfection of cultured sensory neurons with RKIP‐specific siRNA downregulated RKIP expression in its un‐labeled and FITC‐labeled configurations when compared to control (vehicle) and FITC(−) control (Figure 5). Next, FITC‐RKIP siRNA was employed to knock‐down RKIP expression for single‐cell Ca^+2^ imaging. FITC‐positive neurons were exposed to repeated BK (500 nM and 1 μM) to determine whether RKIP influences response desensitization (Figure 6a, b). At both supraphysiological doses, tachyphylactic BK responses decreased in FITC‐RKIP transfected neurons compared to FITC(−) control‐transfected neurons (Figure 6c, d). RKIP knockdown increased 500 nM BK desensitization (67.72 ± 2.41%) compared to FITC(−) control‐transfected neurons (20.89 ± 2.73%). Near‐complete desensitization (92.29 ± 2.26%) in FITC‐RKIP siRNA‐transfected neurons was observed following the second exposure to 1 μM BK, significantly greater than desensitization (48.05 ± 8.57%) observed in FITC(−) control‐transfected neurons. Taken together, these data identify an important role for RKIP in the maintenance of functional responses to the inflammatory mediator BK in sensory neurons.

## 4 DISCUSSION

**FIGURE 7:**
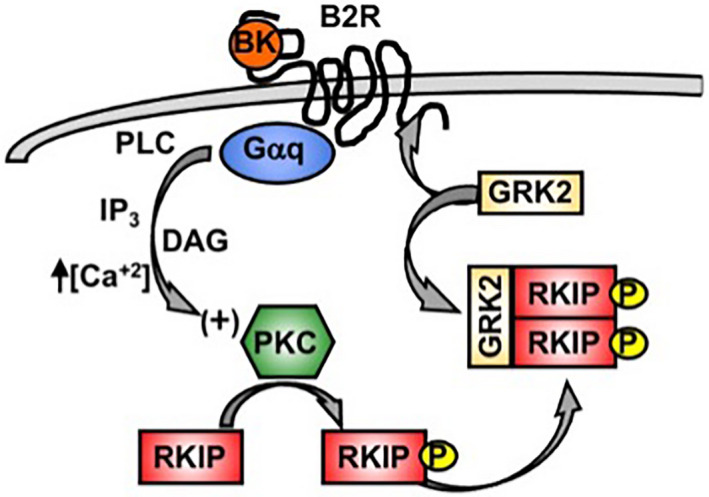
Illustrative diagram of hypothesized signaling mechanism for RKIP‐mediated inhibition of GPCR desensitization

Inflammatory mediators such as BK sensitize primary afferent sensory neurons to nociceptive stimuli via GPCR activation. In most in vitro models, GPCR activation stimulates a canonical internalization event that limits persistent activity. However, persistent inflammatory nociception remains unexplained. Herein, we report that RKIP drives a feed‐forward regulatory system interrupting canonical B2R desensitization to maintain receptor responsivity. As illustrated in Figure 7, BK activation of B2R stimulates Gαq/11‐driven PKC phosphorylation of RKIP at Ser153 to sequester GRK2 and prevent GRK2‐dependent facilitation of agonist‐dependent B2R desensitization.

In an equimolar system, downstream signaling would be considered concentration‐dependent throughout, as evidenced by PKC activity assay results in Figure 2. However, Ca^+2^ imaging results in Figure 4 indicate that secondary signaling pathways demonstrate differential variability in pharmacologic desensitization patterns. Future studies should include physiological concentrations of BK lower than 50 nM, which would be expected to provide even greater sensitization in the absence of RKIP. However, it cannot be presumed that all downstream and regulatory signaling components exist equimolar to receptors, thereby influencing the interpretation that receptor occupancy directly dictates receptor desensitization. Furthermore, the limitations of in vitro experimentation prevent us from modeling tachyphylactic agonist exposure as it likely exists in vivo, as a constant, non‐pulsatile exposure. Nevertheless, our approach highlights an important regulator of GPCR responsivity in a manner unexplained by transfected cell models and supraphysiological agonists.

Supraphysiological treatment with BK (1‐100 μM) rapidly desensitizes B2R in transfected (Blaukat et al., 1996; Fathy et al., 1999) and immortalized cell models (Smith et al., 1995; Wolsing & Rosenbaum, 1991) and is supported by our own observations (Figure 4). However, this range of concentrations neither models human inflammation (Hargreaves et al., 1988a; Hargreaves & Costello, 1990) nor supports behavioral desensitization to acute nanomolar BK injections (Adachi & Ishii, 1979; Miao et al., 1996; Tonussi & Ferreira, 1997). However, a limitation of this study is the lack of consequent data on Store‐Operated Calcium Entry (SOCE), such that following ER depletion of internal calcium stores, BK may induce calcium influx from the extracellular space. Indeed, BK‐stimulation of Orai1 produces transient increases in intracellular Ca^+2^ in sensory neurons (Szteyn et al., 2015), which could inflate outcome measures and confound direct interpretation. Another limitation includes the stability of BK in solution, given its sensitivity to degradation by peptidases including EP24.15 and EP24.16 expressed by sensory neurons (Jeske et al., 2006). Experiments reported here did not utilize peptidase inhibitors throughout Ca^+2^ imaging, resulting in under‐reporting apparent BK concentrations. Future studies will account for these unintentionally biases and contribute more to the physiological relevance of this signaling phenomenon.

A number of GPCRs are negatively modulated by GRK2, supporting the possibility that RKIP sequestration of GRK2 could affect other receptor systems. Recent research identifies roles for RKIP in multiple physiologies (Granovsky et al., 2009), including cancer (Dangi‐Garimella et al., 2009), inflammation (Wright & Vella, 2013), and cardiovascular function (Lorenz et al., 2003). Furthermore, tissue‐specific differences in GRK2/GPCR interactions, such as constitutive DOR/MOR association with GRK2 sensory neurons (Brackley et al., 2016; Zhang & Jeske, 2020) could increase RKIP control of peripheral opioid analgesic efficacy. However, GRK2 interactions with non‐GPCR proteins, including Akt and p38 (Penela et al., 2010) complicate experimental interpretations, especially in terms of disease models and translational relevance. Taken together, the results presented here identify a role for RKIP inhibition of BK receptor desensitization in sensory neurons and suggests a contributory role toward persistent inflammatory hyperalgesia. Future studies should focus on other GPCRs affected by RKIP to explore the centralized utility of RKIP scaffolding and sequestration of GRK2.

## CONFLICT OF INTEREST

The authors have no conflict(s) of interest to declare.

## AUTHOR CONTRIBUTIONS

SBC, ADB, and NAJ performed the experiments and data analysis. SBC, ADB, and NAJ wrote and edited the manuscript. NAJ was responsible for overall project supervision.

### OPEN RESEARCH BADGES

This article has been awarded Open Materials Badge for making the components of the research methodology needed to reproduce the reported procedure and analysis available. More information at Open Science Framework

## Supporting information


Figure 1

## Data Availability

The data that support the findings of this study are available from the corresponding author upon reasonable request.
