# Supplementary material for: Raf kinase inhibitory protein reduces bradykinin receptor desensitization
Source: J Neurochem. 2022 May 8;162(2):156–65. doi: 10.1111/jnc.15614 (PMC9283312; doi:10.1111/jnc.15614)

Title: Raf Kinase Inhibitory Protein Reduces Bradykinin Receptor Desensitization

Running Title: RKIP modulates B2R Function

*Samuel B. Chivers, MS^1^, *Allison Doyle Brackley, PhD^2^, Nathaniel A. Jeske, PhD^1-3^

Supplemental Information

**Supplemental Figure 1.** Full sample Western blot for data generated cumulatively for Figure 3A.

**Supplemental Figure 2.** Full sample Western blot for data generated cumulatively for Figure 3B.

**Supplemental Figure 3.** Full sample Western blot for data generated cumulatively for Figure 5.

Supplemental Figure 1


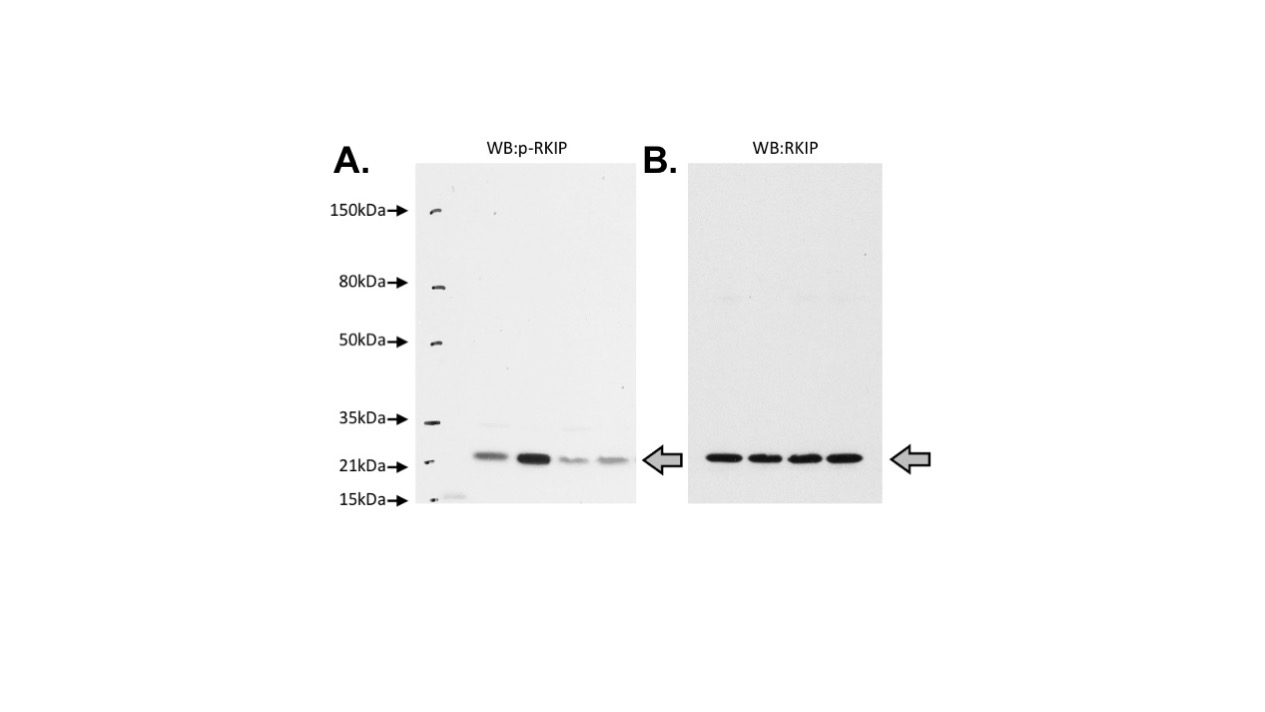


Supplemental Figure 2


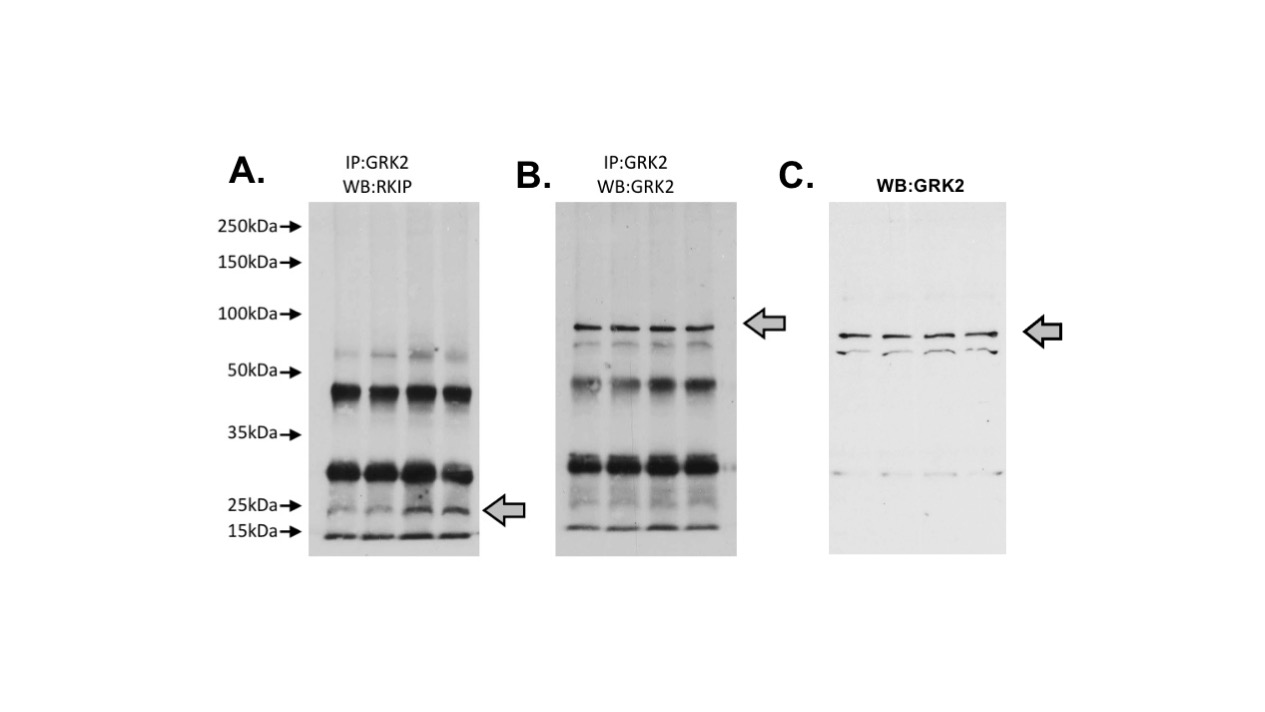


Supplemental Figure 3


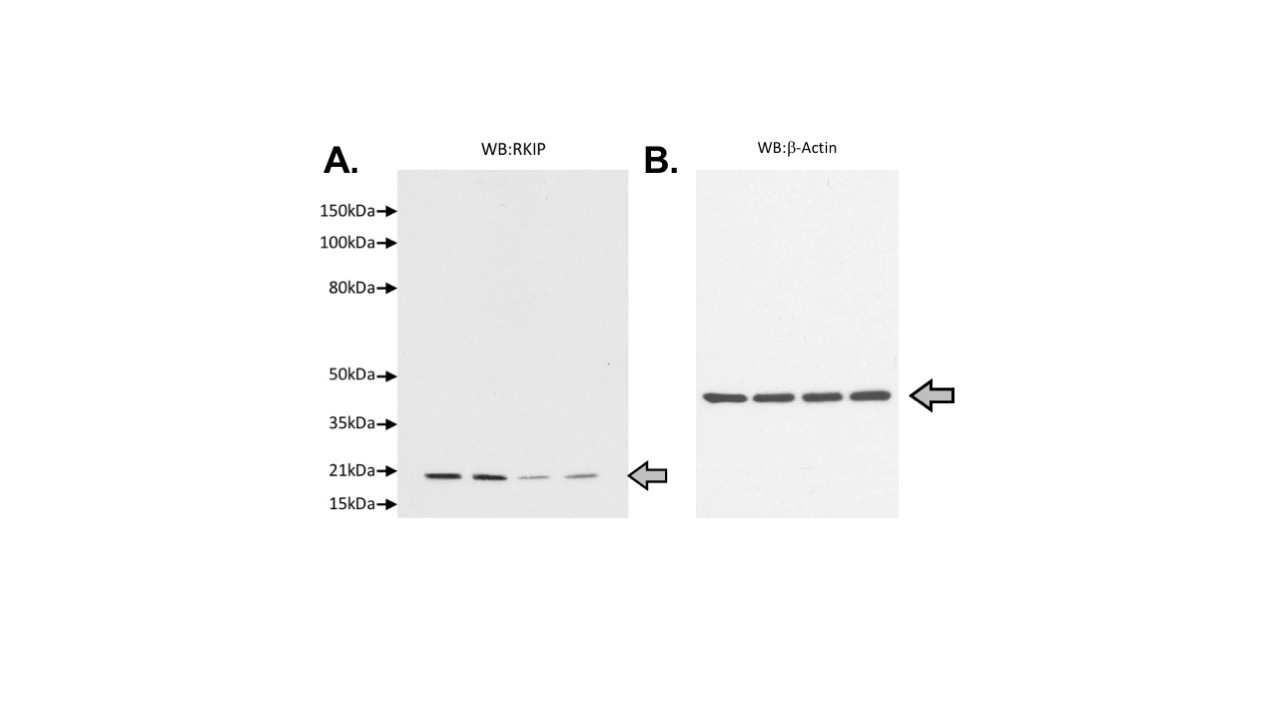

Supplement: Supplementary file 1 — Figure 1 [file JNC-162-156-s001.docx]
